# Enhanced radiative cooling with *Janus *optical properties for low-temperature space cooling

**DOI:** 10.1515/nanoph-2023-0641

**Published:** 2024-01-15

**Authors:** Meng Yang, Yijun Zeng, Qingyuan Du, Haoyang Sun, Yingying Yin, Xiantong Yan, Mengnan Jiang, Chin Pan, Dazhi Sun, Zuankai Wang

**Affiliations:** City University of Hong Kong, Hong Kong SAR, China; Southern University of Science and Technology, Shenzhen, China; The Hong Kong Polytechnic University, Hong Kong SAR, China

**Keywords:** radiative cooling, *Janus* optical property, electrospinning, surface cooling, space cooling

## Abstract

Passive daytime radiative cooling that could provide sub-ambient cooling emerges as a promising technology to reduce household energy consumption. Nonetheless, prevailing studies are predominantly focused on surface cooling, often overlooking its adaptability to enclosed spaces with active cooling technologies. Here we present a multilayer radiative cooling film (*J*-MRC) with *Janus* optical properties in the mid-infrared region, consisting of the nanoporous polyethylene films, the polyethylene oxide film, and silver nanowires. The top side of the *J*-MRC functions as a conventional radiative cooling material to supply sub-ambient surface cooling, while the bottom side with low mid-infrared emissivity transfers limited heat via thermal radiation to the low-temperature enclosures. Our experiments validate that the *J*-MRC possesses an enhanced space cooling performance in comparison to the conventional radiative cooling film. This work provides a valuable design concept for radiative cooling materials, thereby expanding their practical scenarios and contributing to reduce the carbon emission.

## 1 Introduction

Energy stands as an indispensable force driving human production and daily life. However, in present-day society, the energy crisis has become an unavoidable concern, spurred by insufficient energy reserves, and escalating global energy consumption. Apart from diversifying energy sources and harnessing renewable energy, researchers also actively advocate new technologies that can reduce energy demand to confront the energy crisis. As the major component of household energy consumption, conventional refrigeration methods, encompassing air conditioners and refrigerators, consume substantial amounts of electricity for cooling. To decrease the energy requirements for household cooling, passive daytime radiative cooling (PDRC), as a new electricity-free cooling technology, was proposed and extensively studied. PDRC could provide cooling for building [1]–[4] and human body [5]–[7], thus saving cooling energy typically consumed by traditional refrigeration methods. Over the past few years, PDRC was developed by leaps and bounds, both in terms of available raw materials [8]–[12] and preparation techniques [13]–[17].

Generally, daytime radiative cooling materials are designed to significantly reflect sunlight (0.3–2.5 μm) and emit thermal emission through the atmospheric transparent window (8–13 μm) to outer space (∼3 K), thus achieving net outgoing heat flux for cooling. To obtain high solar reflectivity, polymer-based daytime radiative cooling materials are commonly prepared via utilizing the composition with impedance mismatch [18]–[20] and low solar absorptance [21]–[23]. Such composition enables numerous light-scattering interfaces, resulting in a strong scattering effect or diffusive reflection effect [24]. For polymer-based radiative cooling materials, the adopted composition mainly includes polymer and micro/nanoparticle [25]–[28], or polymer and air voids [16], [29]–[31]. Between them, combining polymer with air voids to constitute a porous structure rich in polymer-air interfaces is a more commonly used method due to lower cost and better optical performance [13]. Electrospinning is a well-established method for generating such porous structures, which consist of micro/nanofibers and has plenty of polymer-air interfaces that can scatter sunlight efficiently. Additionally, the electrospinning method is also advantageous in various material selection and adjustable structure parameters [32]. Therefore, adopting the electrospinning method is conducive to extending the scope of radiative cooling applications. However, the mechanical stability of electrospinning membranes presents a noteworthy challenge. Electrospinning membranes, made of micro/nanofibers that lack crosslink, are prone to deformation under mechanical load, which severely limits their practical use. Consequently, improving the mechanical property of electrospinning membranes can definitely increase their applicability for radiative cooling applications.

Aside from achieving high solar reflectivity, possessing high emissivity within the mid-infrared (MIR) region is another indispensable characteristic for radiative cooling materials in order to achieve efficient cooling. Interestingly, most polymers are exceptionally well-suited as candidates for radiative cooling materials as their functional groups have strong absorption within the MIR region, including C–O, C–F, C–N, Si–O and so on [33]–[35]. Then polymer-based radiative cooling materials with high MIR emissivity can utilize radiative heat exchange with outer space to provide cooling. However, an important but commonly overlooked point pertains to bidirectional thermal radiation emitted by radiative cooling materials, which is not only towards outer space, but also facing the underlying enclosures. Therefore, when the internal temperature of enclosures is lower than the temperature of radiative cooling materials, the thermal radiation toward the enclosures will exacerbate the cooling load of the enclosures. During such instances, the radiative heat exchange between the enclosures and radiative cooling materials exerts a counterproductive effect for saving cooling energy. Minimizing the MIR radiative heat exchange between such enclosures (active air-conditioning houses, cold shipping containers, etc.) and radiative cooling materials therefore is necessary for cooling saving, even though radiative cooling materials have shown sub-ambient surface cooling performance.

Here we engineered a flexible radiative cooling film with *Janus* optical properties within the MIR region: high MIR emissivity on the top side and low MIR emissivity on the bottom side. This developed film provides efficient radiative cooling due to high solar reflectivity and MIR emissivity of the top surface, simultaneously reducing radiative heat release towards the cold enclosure, which can decrease the cooling load of the cold enclosure. In addition, compared with the pristine electrospinning film, the developed radiative cooling film has an improved mechanical property because it is densified by the hot-pressing process and further strengthened by polyethylene (PE) films. Practical application tests manifest that the developed radiative cooling film can achieve a comparable sub-ambient surface cooling performance and a better space cooling performance for low-temperature enclosures when compared with the conventional radiative cooling film. The exploratory yet impactful design demonstrates great advantages in developing high-performance passive radiative cooling techniques for space cooling for low-temperature enclosures.

## 2 Materials and methods

### 2.1 Fabrication of electrospinning PEO film

First, PEO powder (*M*_w_ = 600,000 g/mol) and deionized water were mixed at 50 °C under magnetic stirring for about 4 h to obtain 4 wt% PEO solution. After mixing, the obtained solution was stood for 2 h before being used as the electrospinning dope. Then, the electrospinning dope was transferred into a syringe equipped with an 18-gauge stainless steel needle. The electrospinning film was prepared at a voltage of 25 kV, a spinning distance of 20 cm and a feed speed of 1  ml h^−1^. Besides, the electrospinning film was collected by using an aluminium foil that was fixed on a rotating cylinder with a rotation speed of 100 rpm. During the electrospinning process, the temperature and humidity were controlled at 30 °C and 25 %. The finally obtained electrospinning film is called the es-PEO film.

### 2.2 Preparation of the radiative cooling film

The radiative cooling film was prepared by a hot-pressing method. First, commercially available nanoporous PE film (nano-PE, 8 cm × 8 cm) was placed on a hot plate at 50 °C. Concurrently, a specific volume (1.6 ml, 3.2 ml, 4.8 ml, and 6.4 ml) of purchased silver nanowires (AgNWs) solution with a concentration of 10 mg ml^−1^ was diluted to 20 ml using absolute ethanol. Subsequently, the diluted AgNWs solution was slowly and evenly sprayed on commercial PE film to create AgNWs/PE laminated film. Next, AgNWs/PE laminated film, electrospinning PEO film and another commercial PE film were sequentially placed from bottom to top. The resulting multilayer film was then transferred within a hot-pressing mould comprising two stainless steel plates. During the hot-pressing process, the multilayer was first pressed under a pressure of ∼1 MPa at 60 °C for 30 s and then pressed under a pressure of ∼0.3 MPa at 60 °C for 30 min to obtain a dense radiative cooling film, named the *J*-MRC film. Besides, a conventional radiation cooling film prepared using the same method but lacking the AgNWs layer is created as the control sample.

### 2.3 Characterization

Scanning electron microscope (SEM, TESCAN MIRA3) at an accelerating voltage of 10 kV was used to observe the morphologies of the sample. The distribution of pores in PEO film after the hot-pressing process was gained by using Nano Measure software. Fourier transform infrared spectrometer equipped with a gold integrating sphere (FTIR, Bruker Vertex 70v) was utilized to measure the spectral reflection and transmission of the samples within the MIR region (3–16 μm). UV-Vis-NIR spectrophotometer with an integrating sphere (PerkinElmer Lambda 950) was applied to measure the spectral reflection of the samples in the solar region (0.3–2.5 μm). The infrared (IR) images were captured by a thermal infrared camera (FOTRIC 226s). The detection wavelength range of the thermal infrared camera is 8–14 μm. The mechanical property was evaluated by a universal tensile testing machine (Zhiqu ZQ990).

### 2.4 Outdoor cooling performance tests

Outdoor cooling performance measurements for the sample and the contrast sample were conducted in Shenzhen, China by using a homemade apparatus, which can reduce the heat gain from the environment that disturbs the experimental results. The apparatus was constructed from polyethylene (PS) foam (50  cm × 30  cm × 15 cm) covered with aluminium foil to enhance spectral reflection. Within the PS foam, a chamber (18 cm × 10 cm × 7 cm) was made, featuring a window (8 cm × 8 cm) on the top side. During the measurements, the samples were placed on the window to face the sky directly. By attaching PT100 temperature probes on the bottom centre of the samples, the real-time temperatures of the samples were contentiously monitored. The real-time temperatures of the chambers were measured by hanging the temperature probes in the centre of the chambers. The temperature probes before using will be calibrated to ensure the measured temperature difference is no more than 0.1 °C. The ambient temperature was obtained by exposing the PT100 temperature probe to the ambient air, while solar irradiance was collected by using a solar power meter.

## 3 Results and discussion

### 3.1 The design and preparation of the *J*-MRC film

**Figure 1:**
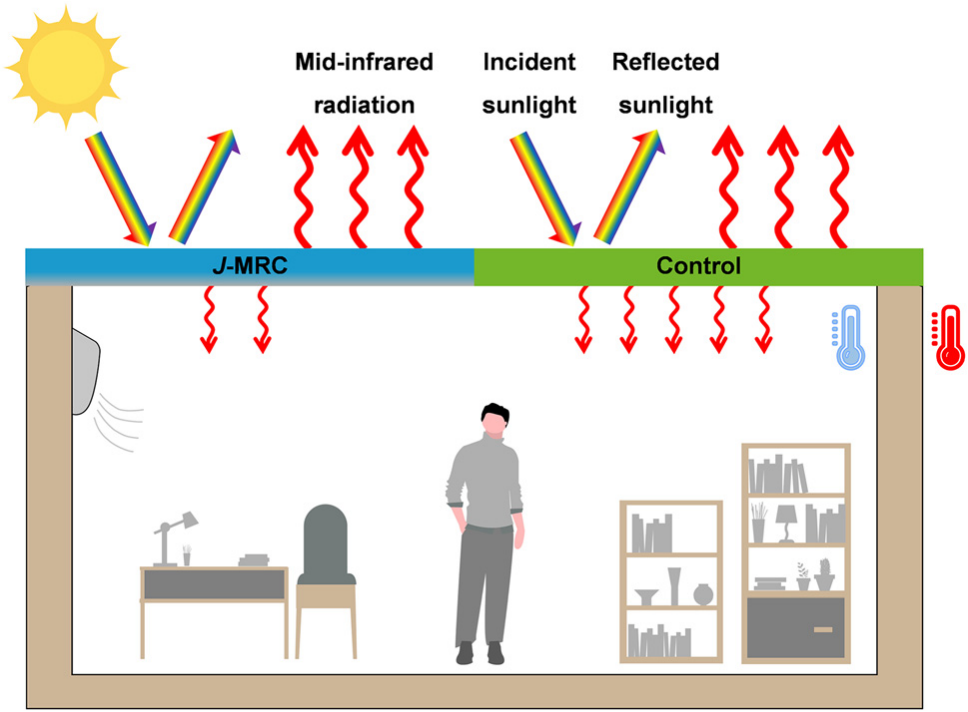
Working principle of the *J*-MRC. Schematic of the *J*-MRC and the control sample (conventional radiative cooler) used as roofs of the enclosure with low inner temperature. Both of them can effectively reflect sunlight and strongly emit infrared thermal radiation to outer space. Different from the control sample, the *J*-MRC only shows high infrared emission in one direction, whose bottom side has high reflectivity and low emissivity in the mid-infrared region, thus reducing radiative heat exchange with underlying low-temperature enclosure.

For the conventional radiative cooling film, exhibiting high solar reflection that diminishes the gain of solar energy and strong MIR emission that enhances thermal loss is necessary, which can generate a net heat loss. Furthermore, to maximize the radiative cooling efficacy, the conventional radiative cooling film is requested to face the sky, so the thermal radiation it emits can easily traverse the atmosphere and then dissipate into outer space. Obviously, in conventional design, only the top side of the film is utilized to provide a cooling effect, while the bottom side is neglected. However, the bottom side that emits inward thermal radiation also has a noteworthy impact on radiative cooling performance. Notably, engineering suitable structures that enable the bottom side for radiative cooling can markedly increase the overall cooling performance [36], [37], or building an emitter featuring the bottom side showing broadband emissivity can more efficiently trap heat from enclosed spaces with extreme heat accumulation [38]. Beyond that, the bottom side can also yield negative effects. For the enclosures with low indoor temperature, the bottom side with broadband emissivity will release heat to the cold enclosures through radiative heat exchange (Figure 1, right part), inevitably escalating the cooling energy consumption of the enclosures.

Given that, we strategically designed the *J*-MRC with *Janus* optical properties for application on cold enclosures (Figure 1, left part). The top side of the *J*-MRC exhibits high solar reflectivity and MIR emissivity, effectively functioning as a conventional radiative cooler. Thus, the top side of the *J*-MRC can provide a continuous cooling temperature below the ambient temperature. Unlike the conventional design with has high MIR emissivity on its bottom side, we customize the bottom side of the *J*-MRC with low MIR emissivity. As mentioned, for special cooling scenarios, like active air-conditioning houses and cold shipping containers, whose inner temperatures are lower than the coolers temperature, the bottom side with low MIR emissivity can reduce the radiative heat release to the cold enclosures. Consequently, the *J*-MRC with *Janus* optical properties can satisfy the special cooling requirements for the cold enclosures that are sub-ambient cooling temperature and limited radiative heat exchange with the hot external environment.

In our design, the top side of the *J*-MRC is composed of a nano-PE film and a hot-pressed PEO film (HP-PEO), which both have porous structures that are rich in light-scattering air voids, thereby enabling efficient sunlight scattering. Besides, the HP-PEO film which has a large amount of C–O–C (1260–1110 cm^−1^) and C–OH (1239–1030 cm^−1^) bonds can emit strongly within the MIR region [8]. Though the nano-PE film shows very limited help with MIR emission due to its high MIR transmittance, it is expected to provide mechanical protection for the HP-PEO film. The bottom side of the *J*-MRC comprises a nano-PE film and an AgNWs layer, securely integrated into the HP-PEO film via the hot-pressing process. Similarly, the nano-PE film could provide mechanical protection and has negligible impact on the infrared optical properties of the AgNWs layer. The AgNWs layer at high usage can isolate the top side and the underlying spaces in the MIR region due to its high MIR reflectivity and low MIR transmission [39]. Therefore, the AgNWs layer can significantly curtail the radiative heat transfer from the *J*-MRC to the cold enclosures.

**Figure 2:**
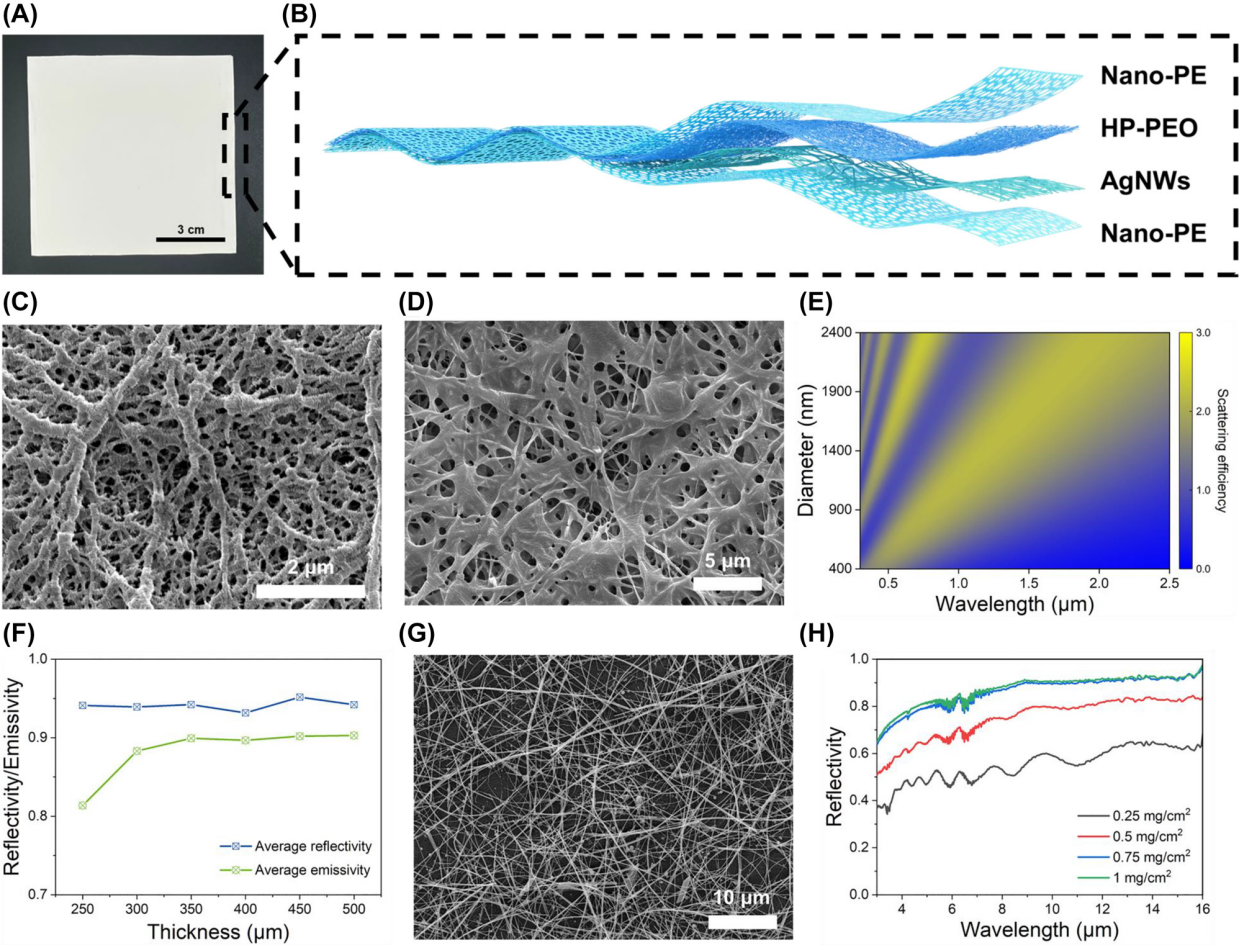
Structure of the *J*-MRC. (A) Optical photograph of the *J*-MRC. (B) Schematic of the microstructure of the *J*-MRC, composed of two nano-PE layers, an HP-PEO layer and an AgNWs layer. (C) SEM image of the nano-PE layer. (D) SEM image of the HP-PEO layer. The layer has a crosslinked network microstructure after the hot-pressing process. (E) Scattering efficiency of pores with different diameters in the HP-PEO matrix. (F) Average solar reflectivity and average emissivity in the atmospheric transparent window of the HP-PEO layers with different thicknesses. (G) SEM image of the AgNWs layer sprayed on the nano-PE film. (H) Reflectivity spectra in the mid-infrared regions of the AgNWs layer with different AgNWs dosage.

Figure 2A shows the optical photograph of the as-prepared *J*-MRC film, exhibiting a white colouration. As conceptually demonstrated in Figure 2B, the *J*-MRC film consists of four functional layers. The first and last layers are both the nano-PE films which can scatter a portion of incident sunlight and protect the internal HP-PEO film from deformation. As shown in Figure 2C, the nano-PE film has plentiful nanopores, whose sizes are comparable to the wavelength of ultraviolet and visible light, thus showing relatively high reflectivity in the corresponding region (Figure S1).

The second layer is the HP-PEO film. Same with other similar work [40], the es-PEO film is composed of abundant nanofibers with the presence of beads that formed on these nanofibers (Figure S2). During the hot-pressing process, the nanofibers and beads are melted and extruded to form a porous network structure (Figure 2D). Different from the fibrous structure, the porous network structure is thought to utilize air voids to scatter sunlight. We measured the size of the pores in the HP-PEO film. The results indicate that the size of pores has a broad distribution from 400 nm to 2400 nm and centres at approximately 800 nm (Figure S3). We then employ finite-difference time-domain (FDTD) simulations to investigate the scattering efficiency of pores in the HP-PEO film (Supplementary Method S1). As illustrated in Figure 2E, nanopores can only strongly scatter ultraviolet and visible light, while micropores can scatter light in the whole solar spectrum. The combined effect of nanopores and micropores endows the HP-PEO film with high solar reflectivity.

To obtain the highest solar reflectivity for the top side of the *J*-MRC, we measured the spectral reflectivity of different top-side films in the solar wavelength range, which consisted of the nano-PE films and the HP-PEO films with different thicknesses (Figure S4A), and then calculated their average solar reflectivity ( ${\bar{R}}_{\mathrm{solar}}$ ) (Supplementary Method S2). Benefitting from substantial micro/nanopores in the nano-PE films and HP-PEO films, all the top-side films show high solar reflectivity above 0.93 (Figure 2F). In addition, we also measured the spectral reflectivity and transmittance of different top-side films within the MIR region, and then determined the spectral emissivity based on Kirchoff’s law (Figure S4B). Clearly, the emissivity increases as the top-side films thicken. We then calculated the average emissivity ( ${\bar{\varepsilon }}_{\mathrm{LWIR}}$ ) of the top-side films in the wavelength range of 8–13 μm (Supplementary Method S3). The results declare that the ${\bar{\varepsilon }}_{\mathrm{LWIR}}$ reaches the highest value as the thickness increases to 350 μm, which has considerable emissivity with the thicker films (Figure 2F). Based on the above discussion, we decided to utilize a 350-μm top-side film to prepare the *J*-MRC.

The third layer is the AgNWs layer, which is embedded on the surface of the HP-PEO film after the hot-pressing process (Figure S5). To ascertain the optimal AgNWs dosage, we characterized the MIR optical properties of the AgNWs layers, which were prepared via spraying AgNWs solution on the nano-PE films. The reflectivity and transmission spectra of the AgNWs layers in the MIR region are shown in Figures 2H and S6, respectively. Apparently, the MIR reflectivity shows an upward trend with increasing AgNWs dosage, eventually reaching near-maximum values at a dosage of 0.75 mg cm^−2^. The transmission spectra exhibit an opposite trend that is the MIR transmission decreases as the dosage increases. Similarly, the MIR transmission gets a near-minimum value when the dosage is 0.75 mg cm^−2^. For cost considerations, we opted to employ the AgNWs layer with the dosage of 0.75 mg cm^−2^ for further preparing the *J*-MRC. Figure 2G presents the SEM image of the employed AgNWs layer, demonstrating an AgNWs network with submicron spacing. Such AgNWs layer with high MIR reflectivity and low MIR transmission endows the bottom side of the *J*-MRC with low MIR emissivity, reducing the radiative heat exchange with the underlying enclosure.

### 3.2 The characterizations of the *J*-MRC film

**Figure 3:**
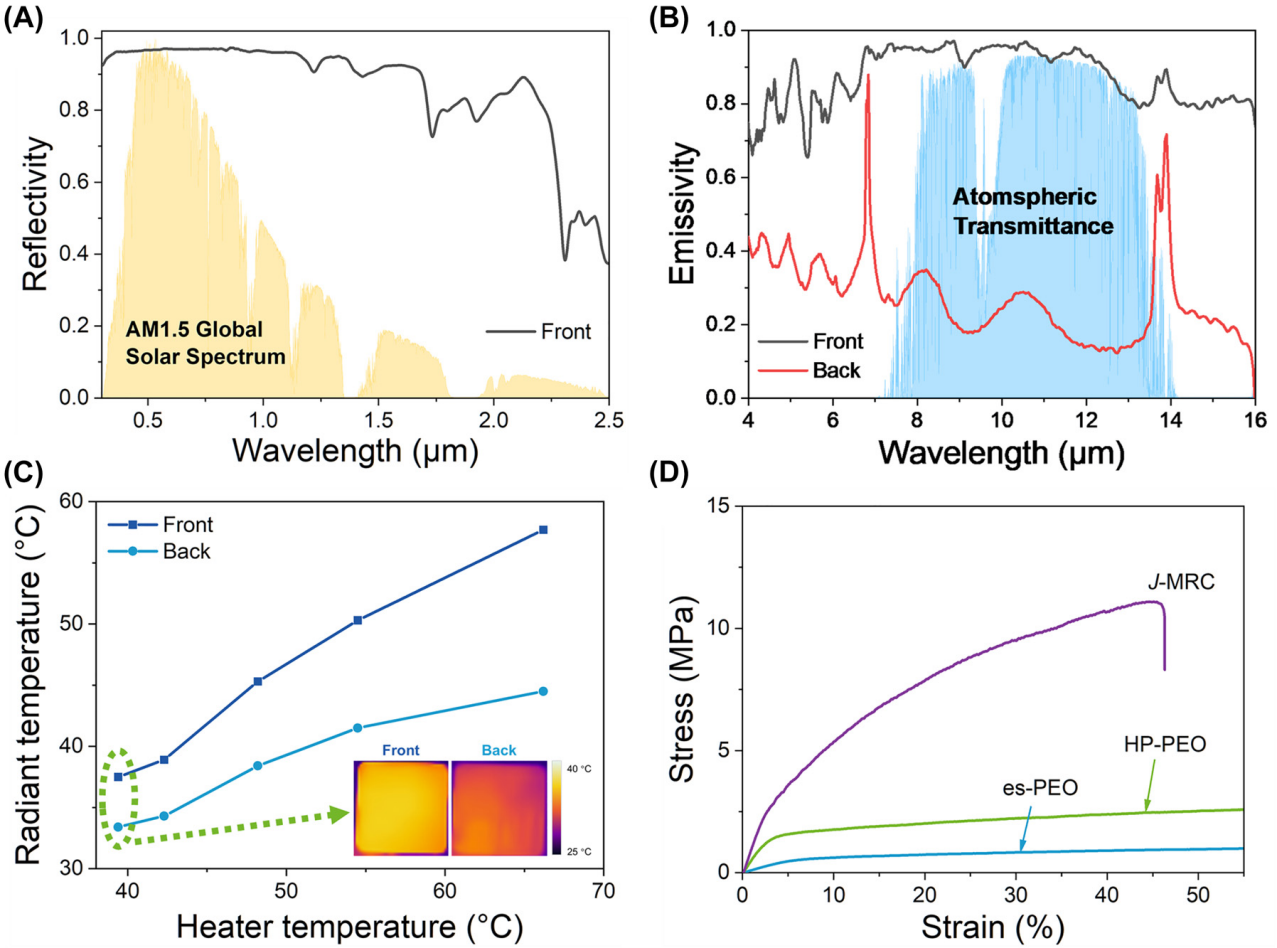
Characterization of the *J*-MRC. (A) Reflectivity spectrum in the UV-Vis-NIR wavelength range measured from the front of the *J*-MRC. The yellow shaded areas represent the AM1.5 global solar spectrum for reference. (B) Emissivity spectra in the mid-infrared regions measured from the front and back of the *J*-MRC. The blue shaded areas show the atmospheric transmittance for reference. (C) The radiant temperature of the front and back of the *J*-MRC on the heater with different temperatures. Inset: infrared images of the front and back of the *J*-MRC on the heater at 39 °C. (D) Stress-strain curves of the es-PEO film, HP-PEO film, and *J*-MRC film.

We further characterized the spectral reflectivity and emissivity of the *J*-MRC to illustrate its *Janus* optical properties. To clearly distinguish, we defined the spectral measurements taken from the top side and the bottom side as the front spectrum and the back spectrum, respectively. As depicted in Figure 3A, the reflectivity spectrum of the front verifies that the porous structure of the nano-PE film and the HP-PEO film can efficiently scatter sunlight, thus leading to a high ${\bar{R}}_{\mathrm{solar}}$ of ∼0.954. The emissivity spectra within the MIR region are found with significant difference when measured from different sides (Figure 3B). The obtained MIR emissivity spectrum of the front has an average value of ∼0.932 in the wavelength region of 8–13 μm, meaning that the top side of the *J*-MRC can strongly dissipate heat through the atmospheric transparent window. In contrast, the obtained MIR emissivity of the back is notably low, which has an average value of ∼0.215 in the same 8–13 μm wavelength range. The low emissivity of the back can be attributed to the high MIR reflectivity and low MIR transmission of the AgNWs layer. The spectral result underscores the fact that the bottom side of the *J*-MRC can only emit limited thermal radiation, same as limited radiative heat exchange between the *J*-MRC and the underlying enclosure.

To visualize the contrasting infrared radiation between the two sides of the *J*-MRC, we placed the *J*-MRC on the heater with a stable temperature and monitored the radiant temperature of the *J*-MRC by using an infrared thermal camera. We defined the temperature when the top side faces the camera as the front temperature and the temperature when the bottom side faces the camera as the back temperature. As shown in Figure 3C, the radiant temperature of the front and back all have a clear difference regardless of the heater temperature. For instance, the radiant temperature of the front and the back are ∼37.5 °C and 33.4 °C while the heater temperature is set at 40 °C. The low radiant temperature of the back implies the radiative heat release of the bottom side is low. Moreover, the radiant temperature difference becomes more extensive as the heater temperature increases, which is due to the difference in the MIR emissivity between the two sides. Apart from purposefully designed infrared optical properties of the two sides, the *J*-MRC also has enhanced mechanical property comparing to the bulk electrospinning membrane, which renders it well-suited for practical applications. As illustrated in Figure 3D, the es-PEO film has the weakest tensile strength and the *J*-MRC film has the highest one. Through the hot-pressing process, the HP-PEO film is strengthened due to the formed crosslinked network structure. By incorporating the nano-PE film, the tensile strength can be further strengthened. The tensile strength of the *J*-MRC is ∼11 MPa, marking a substantial improvement of several times over the es-PEO film.

### 3.3 Outdoor cooling performance tests

**Figure 4:**
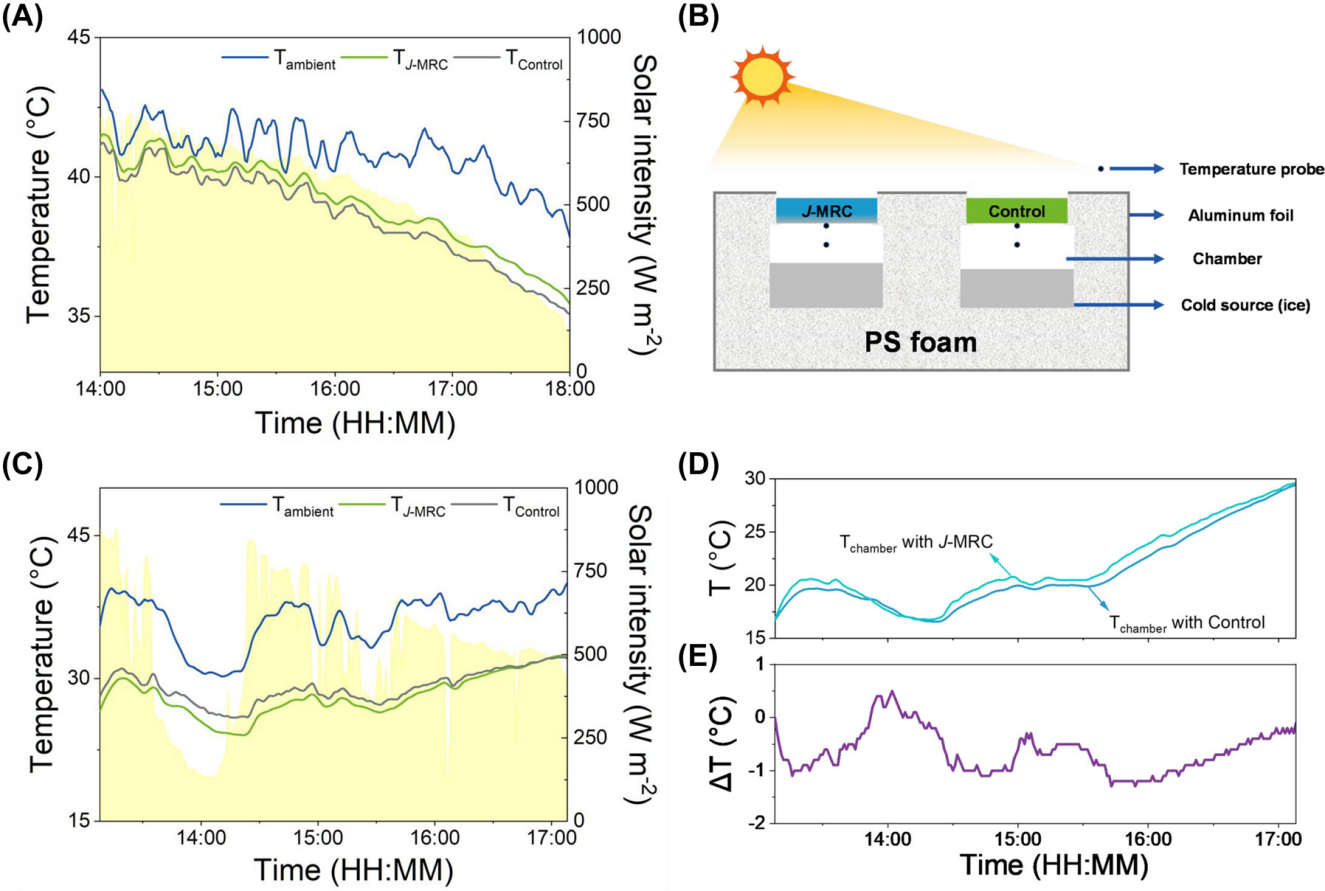
Surface and space cooling performance of the *J*-MRC. (A) Temperature of the ambient air, *J*-MRC and control in outdoor surface cooling performance test (July 27, 2023). The yellow shaded areas represent the measured solar intensity during the test. (B) Schematic of the homemade apparatus for space cooling performance test under sunlight. (C) Temperature of the ambient air, *J*-MRC and control in outdoor space cooling performance test (August 3, 2023). The yellow shaded areas represent the measured solar intensity during the test. (D) The temperature comparisons between the chambers covered by the *J*-MRC and control in outdoor space cooling performance. (E) The temperature difference of the chambers covered by the *J*-MRC and control.

The surface cooling performance test was carried out on a clear day via using a homemade apparatus (Figure S7). The local wind speed and humidity during the test are shown in Figure S9. The high solar reflectivity and MIR emissivity ensure the *J*-MRC film and control film to achieve efficient daytime sub-ambient cooling performance (Figures 4A and S8). Exposing under direct sunlight, the temperature of the *J*-MRC film and control film are both lower than the ambient temperature. At 14:00, a time when the highest solar irradiance commonly appears, the sub-ambient temperature drops of two coolers are relatively small. This is because the coolers absorb large amounts of solar energy at high solar irradiance, consequently diminishing their net cooling performance. With time going on and solar intensity decreasing, the sub-ambient temperature drops become bigger. Besides, the temperature of the *J*-MRC film is consistently higher than that of the control film. This phenomenon is incomprehensible because the top side of *J*-MRC film has higher ${\bar{R}}_{\mathrm{solar}}$ and ${\bar{\varepsilon }}_{\mathrm{LWIR}}$ compared with the control film whose ${\bar{R}}_{\mathrm{solar}}$ and ${\bar{\varepsilon }}_{\mathrm{LWIR}}$ are ∼0.942 and ∼0.899, respectively. However, when considering the effect of the bottom side of the *J*-MRC film, this phenomenon becomes reasonable. The bottom side of the *J*-MRC film has a low MIR emissivity, which blocks the thermal radiation to the underlying enclosure, thereby hampering the net cooling performance of the *J*-MRC film [41].

Unlike the surface cooling performance test, the space cooling performance test involved the application of a cold source (∼100-g ice) in the chambers to simulate the enclosures equipped with active cooling technology (Figures 4B and S10). We thus monitored the chambers temperature to study the effect of the coolers. The testing location remained consistent with that of the surface cooling performance test. The local wind speed and humidity during the test are exhibited in Figure S11. As observed, the surface temperature of the *J*-MRC film and control film are both much lower than the ambient temperature, which is attributed to the presence of a cold source that induces a low inner temperature within the chambers (Figure 4C). Interestingly, in contrast to the results of the surface cooling performance test, the *J*-MRC film demonstrates a lower temperature than the control film. This is because the surface temperature of the *J*-MRC and control film covered on the cold enclosures is mainly influenced by the different chamber temperatures.

As demonstrated in Figure 4D, the chamber covered by the *J*-MRC film has a lower internal temperature compared with the one covered by the control film. The difference in the chamber temperature is caused by the different radiative heat release from the above films, considering that they share almost the same external environment. The temperature difference between the two chambers is demonstrated in Figure 4E. As illustrated, the temperature difference can reach a maximum of 1.4 °C when using ∼100-g ice as the cold source. As expected, the *J*-MRC film can simultaneously offer conventional surface radiative cooling and reduced radiative heat release to the cold space, thus maintaining a lower temperature in the chamber under the same cold load as the one covered with the control film. Therefore, compared with the control film prepared in conventional design, the *J*-MRC film exhibits enhanced radiative cooling for the special low-temperature cooling applications.

## 4 Conclusions

As a passive cooling strategy aimed at reducing household energy consumption, the *J*-MRC film was ingeniously designed with Janus optical properties to provide efficient radiative cooling on its top side while minimum radiative heat transfer to the underlying cold enclosure on its bottom side. The top side, encompassing the nano-PE film and the HP-PEO film, shows high solar reflectivity and MIR emissivity, guaranteeing efficient cooling. While the bottom side comprised of the AgNWs layer and nano-PE film presents low MIR emissivity, indicating the limited thermal radiation towards the underlying cold enclosure. The surface cooling performance test suggests that the *J*-MRC actually exhibits a diminished cooling performance compared to the conventional radiative cooling film. However, for the practical applications involving radiative cooling for cold enclosures, the *J*-MRC has more significant advantages in maintaining lower inner temperature within the enclosures. As evidenced by the space cooling performance test, the chamber covered by the *J*-MRC film can be maximumly lower 1.4 °C than the chamber covered by the conventional radiative cooling film. Our work offers a fresh design concept for preparing daytime radiative coolers, no longer limited to surface cooling, but focused on space cooling for low-temperature enclosures.

## Supplementary Material

Supplementary Material Details
