# Supplementary material for: Enhanced radiative cooling with Janus optical properties for low-temperature space cooling
Source: Nanophotonics. 2024 Jan 15;13(5):629–37. doi: 10.1515/nanoph-2023-0641 (PMC11501828; doi:10.1515/nanoph-2023-0641)
Supplement: Supplementary file 1 — Supplementary Material Details [file j_nanoph-2023-0641_suppl_001.docx]

Supplementary Information for

Enhanced radiative cooling with Janus optical properties for low-temperature space cooling

Meng Yang^1,2^, Yijun Zeng^1,3^, Qingyuan Du^2^, Haoyang Sun^2^, Yingying Yin^1^, Xiantong Yan^1^, Mengnan Jiang^3^, Chin Pan^1^, Dazhi Sun^2,*^, Zuankai Wang^3,*^

*^1^Department of Mechanical Engineering, City University of Hong Kong, Hong Kong 999077, PR China*

*^2^Department of Materials Science and Engineering, Southern University of Science and Technology, Shenzhen, Guangdong, 518055, PR China*

*^3^Department of Mechanical Engineering, The Hong Kong Polytechnic University, Hong Kong 999077, PR China*

**Corresponding authors’ email:* [sundz@sustech.edu.cn](mailto:sundz@sustech.edu.cn); [zk.wang@polyu.edu.hk](mailto:zk.wang@polyu.edu.hk)

This file includes:

Supplementary Methods. S1, S2, and S3

Supplementary Figures. S1, S2, S3, S4, S5, S6, S7, S8, S9, S10, S11 and S12

**Supplementary method S1:**

**Finite-Difference Time-Domain (FDTD) simulation**

We used FDTD solutions software by Lumerical to investigate the scattering efficiency of the micro/nanopores in the HP-PEO film to sunlight. A simple two-dimensional model that is pore in homogeneous PEO medium was employed due to the performance limits of computed devices. A total-field scattered-field source (TFSF) and a perfectly matched layer (PML) was applied for simulations. In the solar wavelengths (0.3-2.5 μm), the scattering cross sections of pores with sizes of 400-2400 nm was simulated and then the scattering efficiency was calculated.

**Supplementary method S2:**

**Calculation of average solar reflectivity**

In the solar wavelengths (0.3-2.5 μm), the average solar reflectivity is defined as:

$$\bar{R}_{solar}=\frac{\int_{0.3 \mu m}^{2.5 \mu m} I_{AM1.5}\left( \lambda\right)R\left( \lambda\right)d\lambda}{\int_{0.3 \mu m}^{2.5 \mu m} I_{AM1.5}\left( \lambda\right)d\lambda}$$

where $\lambda$ is the wavelength, $I_{AM1.5}\left( \lambda\right)$ is the AM1.5 Global solar intensity spectrum, and $R\left( \lambda\right)$ is the surface’s spectral reflectivity of the samples.

**Supplementary method S3:**

**Calculation of average emissivity in the atmospheric transparent window**

The average emissivity is obtained by averaging the obtained MIR emissivity with wavelength range of 8-13 μm, which is given by:

$$\bar{\varepsilon}_{LWIR}=\frac{\int_{8 \mu m}^{13 \mu m} I_{BB}\left( \lambda, T \right)\varepsilon\left( \lambda\right)d\lambda}{\int_{8 \mu m}^{13 \mu m} I_{BB}\left( \lambda, T \right)d\lambda}$$

where $I_{BB}\left( \lambda, T \right)$ is the radiation intensity emitted by a blackbody at the absolute temperature T and $\varepsilon\left( \lambda\right)$ is the surface’s spectral emissivity of the samples.

The blackbody radiation intensity $I_{BB}\left( \lambda, T \right)$ is defined as:

$$I_{BB}\left( \lambda, T \right)=\frac{2hc^{2}}{\lambda^{5}}\frac{1}{e^{{hc}/{(\lambda k_{B}T)}}-1}$$

where $h$ is the Planck constant, $c$ is the speed of light in vacuum and $k_{B}$ is the Boltzmann constant.

Supplementary Figure S1. Reflectivity spectrum of the nano-PE film in the UV-Vis-NIR wavelength range.


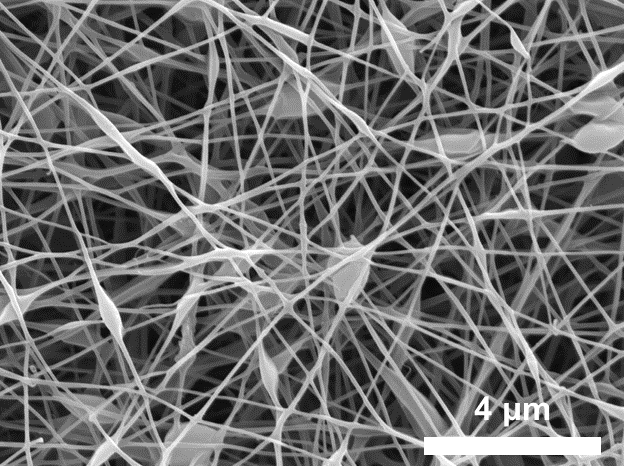


Supplementary Figure S2. SEM image of the es-PEO layer.


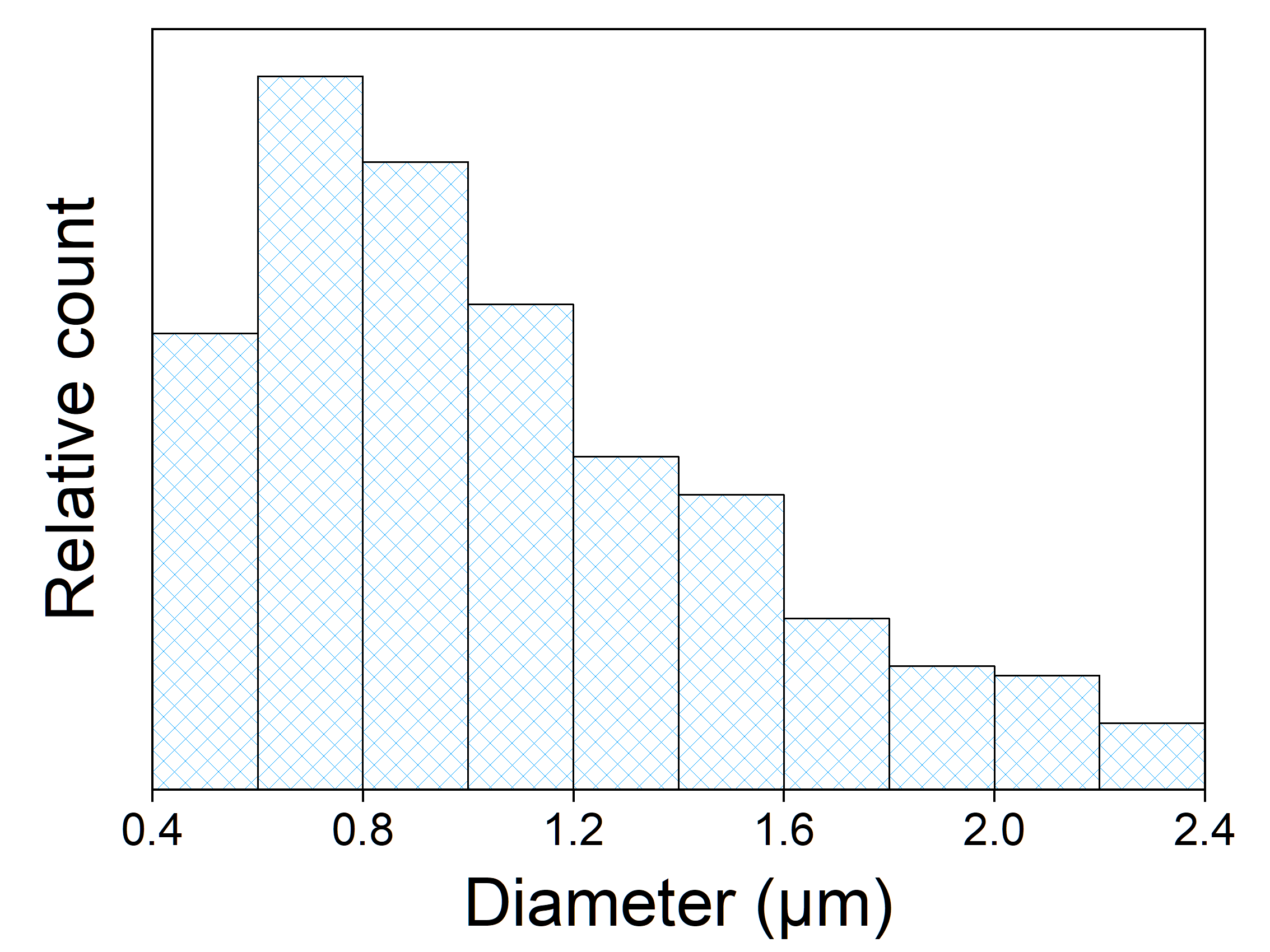


Supplementary Figure S3. Size distributions of pores in the HP-PEO film.


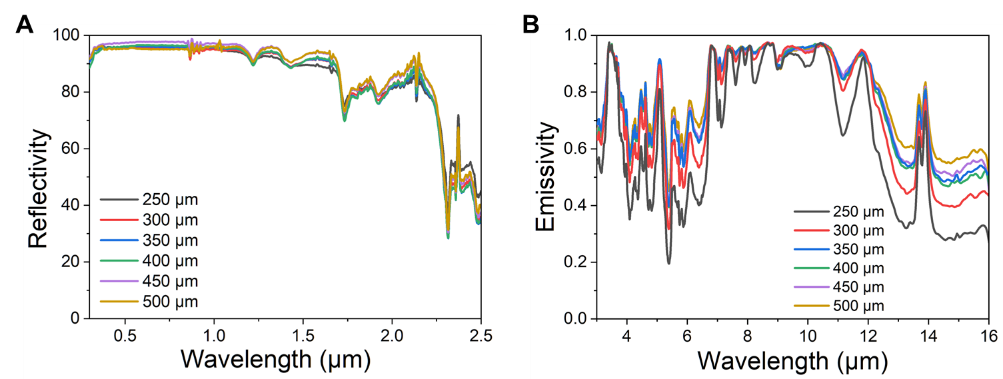


Supplementary Figure S4. (A) Reflectivity spectra in the UV-Vis-NIR wavelength range and (B) emissivity spectra in the mid-infrared regions of the top-side films with different thicknesses.


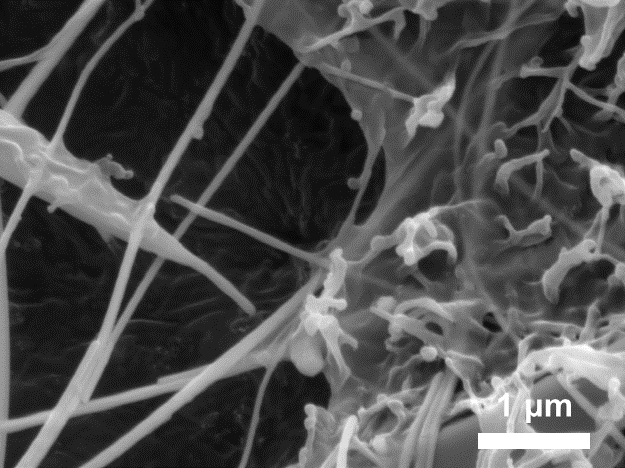


Supplementary Figure S5. SEM image of the AgNWs layers after the hot-pressing process.


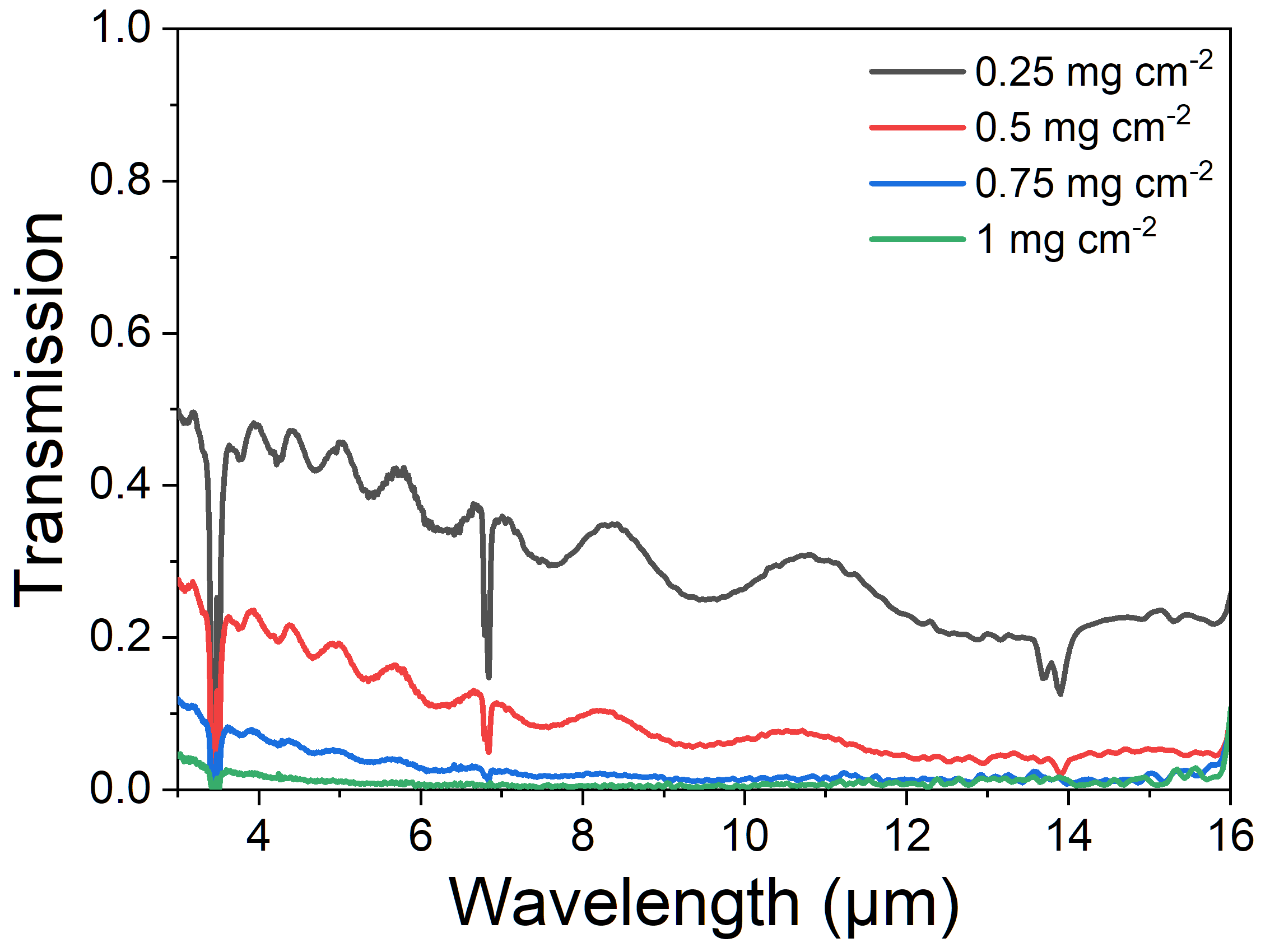


Supplementary Figure S6. Transmission spectra in the mid-infrared regions of the AgNWs layers.


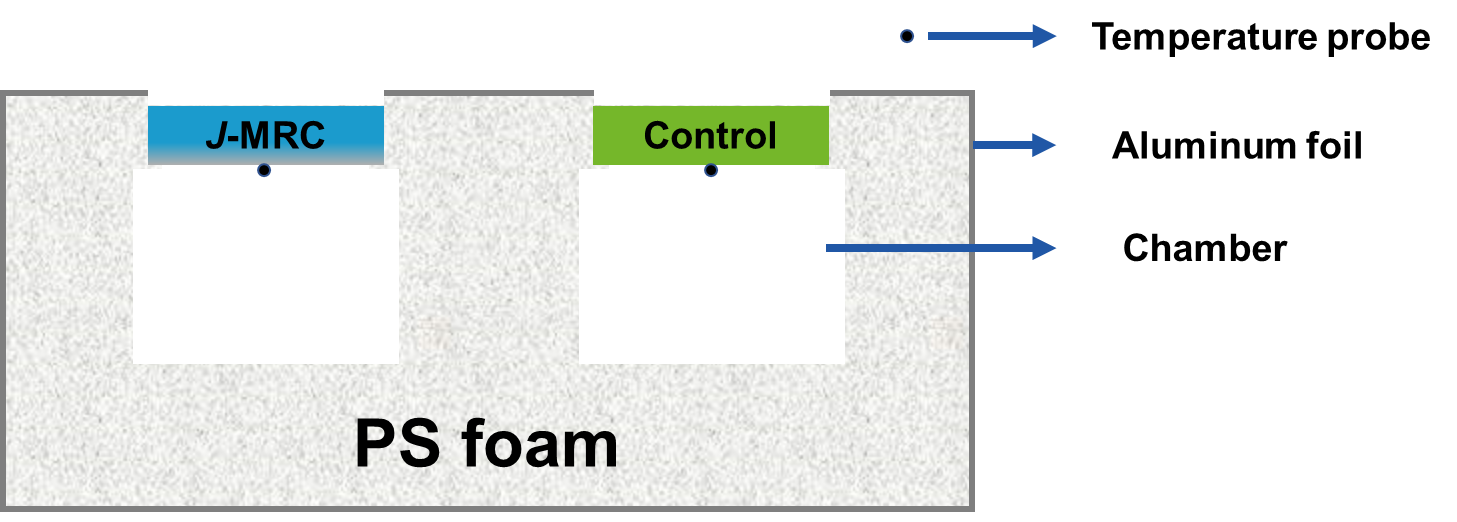


Supplementary Figure S7. Homemade apparatus for the surface cooling performance test.


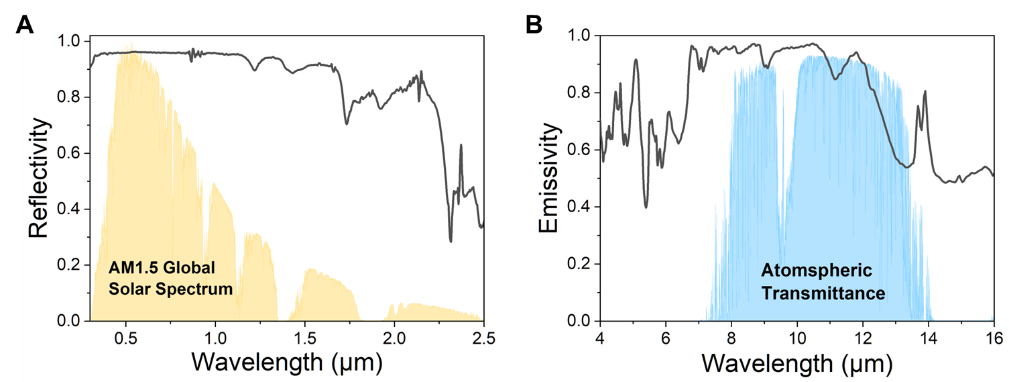


Supplementary Figure S8. (A) Reflectivity spectrum in the UV-Vis-NIR wavelength range measured from the front of the control sample. The yellow shaded areas represent the AM1.5 global solar spectrum for reference. (B) Emissivity spectra in the mid-infrared regions measured from the front and back of the control sample. The blue shaded areas show the atmospheric transmittance for reference.


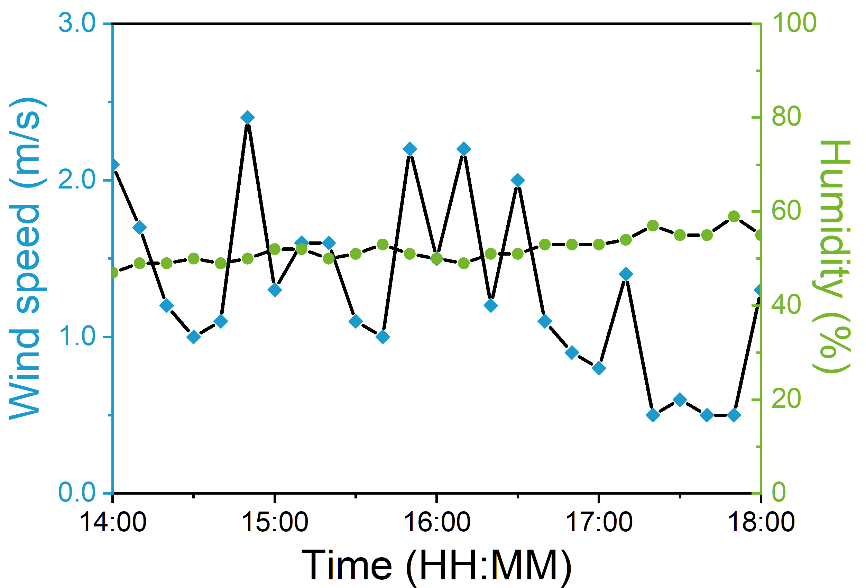


Supplementary Figure S9. Local wind speed and humidity during outdoor surface cooling performance test (July 27, 2023). The data was collected from the website of the Meteorological Bureau of Shenzhen Municipality.


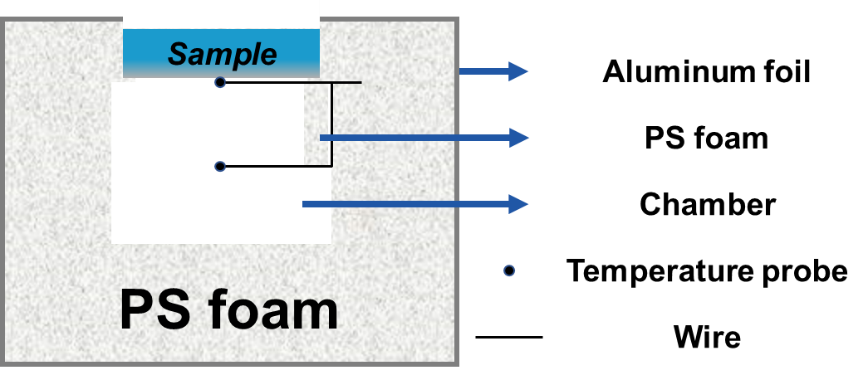


Supplementary Figure S10. Cross section of the homemade apparatus for space cooling performance. The thermocouples were hung in the centre of the chambers by utilizing PS foam to fix most of the thermocouple wires. Considering the mechanical property of metal wires, the wire end with a thermocouple has minimal bending deformation even though a small portion of the wire were not fixed. Thus, the thermocouples can be hung in the centre of the chambers without shelter.

Supplementary Figure S11. Local wind speed and humidity during outdoor space cooling performance test (August 3, 2023). The data was collected from the website of the Meteorological Bureau of Shenzhen Municipality.


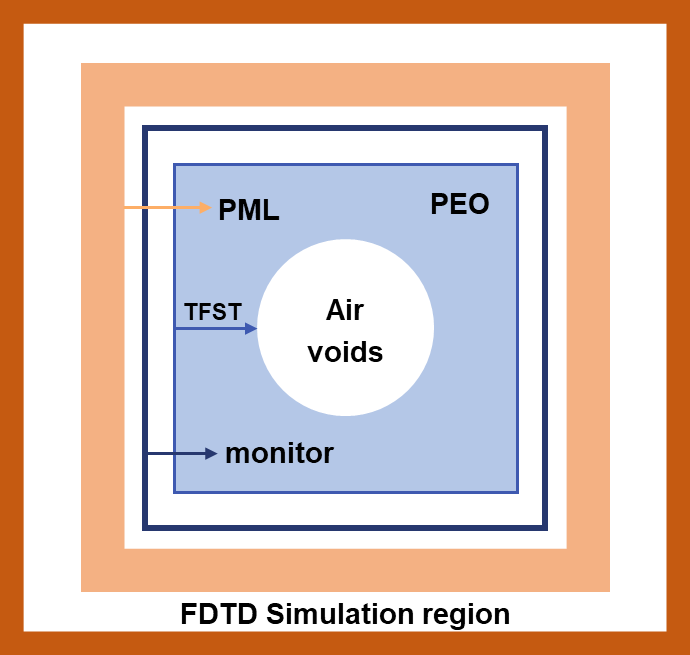


Supplementary Figure S12. The two-dimensional model used in FDTD simulations.
